# The achievement of a storytelling about Chagas disease: how to talk about silence; how to visualise the invisible

**DOI:** 10.1590/0074-02760220083chgsa

**Published:** 2022-07-13

**Authors:** Francisco Javier Sancho Mas

**Affiliations:** 1 Coordinator of Chagas Disease Global Coalition (based in Barcelona, Spain); 2 Former Communicator of Doctors Without Borders (Médécins Sans Frontières, MSF)

Two adjectives raise an issue for communicators working on Chagas disease (CD): “invisible and silent”. Two adjectives that can be ascribed to other neglected tropical diseases (NTD), but which are part of the essence of CD. Bringing CD out of its situation of neglect and oblivion is a mission entrusted mainly to the world of communication as well as of science, politics and financial resources. However, communication has not always been considered among the priorities in the approach to the disease, except in valuable exceptions, some of which we have seen in the preceding article.

In any case, and based on what I have learned from my experience in the area of communication, journalism and advocacy, the main problem lies in the fact that the very essence of communication is weakened, sometimes impotent, applied to a disease that challenges the two main communication tools: images and words. How to speak of silence and how to visualise the invisible? It can be seen as a metaphorical play on words, but it is full of meaning for this disease.

**A matter of seconds, or even faster**

Chagas calls for a communication strategy that serves as a mobilisation tool for the affected communities, countries where the disease is present, decision-makers and global health community. For any communication strategy it is essential to have a clear message. Right after the message, the concern is how to dress and present the message and expand its potential impact. This means to find a symbol.

In advertising, for instance, we usually react in seconds, sometimes in fractions of seconds. Images, gestures and words can inform the audience, as well as to make them move after provoking a desire and the willingness to perform an action or purchase a product. Advertising always aims at maximising time. Let’s think of a single person walking on the street. The short time that elapses between the passerby’s glance at an advertisement in the street and something else catching his/her attention is the available period to communicate a message and impact. Because of the digital era and dynamics of social media this short period of time is even faster. So, the message has to be captured from the first images or words (before the user moves on to the next content offered by his/her channel or account in Tiktok, Facebook, WhatsApp or other social media or APP).

This example from Advertising is similar to what communication faces in the world of NTD. In fact, it faces a wider range of challenges. None of the NTD, not even when their elimination is achieved, occupies the headlines of the mainstream media or the most prominent content of social networks. For people affected by CD, whose illness has been known for more than 100 years, that means invariable invisibility and silence, more than “100 years of solitude”, to borrow the title of the famous book of Gabriel García Márquez. The article “How people affected by Chagas disease have struggled with their negligence: history, associative movement and World Chagas Disease Day” makes an exhaustive, comprehensive and necessary summary of the history of the associations of people affected by the disease.

After two decades attempting to make visible the stories of people affected by humanitarian crises and neglected diseases, Chagas has not yet found the story-message that, in a few words and images, reaches society, drives the action of decision-makers and sufficiently sensitises and interests them all. The process of achieving the final and definitive storytelling about CD is still ongoing. But we are already closer. Some signs allow us to think so.

**People’s voice and communication should be at the early stage of scientific thinking**

It is really important that the Table I “Multidimensional reality of CD with its complex socio-economic, environmental health dimensions”, shown in the preceding article includes “information and communication”. It is emphasised that the lack of information about the disease undermines the right of affected persons to protect and defend themselves. Finally, it also impacts on political disinterest and the lack of demand for care by society and the affected persons themselves.

This minor impact of communication, since the early stages of the knowledge about the disease, is probably parallel to the low interest in science to develop new tools for a disease that, until not long ago, was considered by some health professionals to be just the result of an autoimmune condition. However, as the article details, science has not always forgotten the voice of the people affected, whose strength is essential when it comes to raising the challenges and the demand for answers at the appropriate political level, where decisions are made and resources are raised to meet a public health need.

Another likely problem is that science and communication have neither cooperated sufficiently nor have always cooperated effectively. The Coronavirus disease 19 (COVID-19) pandemic made this challenge evident, again. I think that one of the causes lies in the fact that dissemination, raising-awareness actions and communication strategies are not usually at the beginning of scientific approaches that seek impact results, but are included at the end of them, in a stage very close to implementation or as a complement to the response or design of a research study. It is thus a necessary tool, but an afterthought. On the contrary, I believe that the scientific proposal and the implementation of programs should include a communication component from the first phase of their planning and development, searching for a wide dissemination among the beneficiaries and targeted audience, far beyond one or several scientific publications.

In the preceding article, the first images that Dr Carlos Chagas himself took of the affected people and communities are mentioned as an example of his deep understanding that this was a disease related to social injustice. And likewise, in the letters preserved by one of his disciples, Emmanuel Dias, we can approach the visions and concerns of the affected people at that time when there was not even treatment. Both the images captured by Dr Chagas, as well as the words of the patients preserved by Dr Dias, are a treasure in which to extract lessons that can guide us in the construction of the message-narrative that ends up telling the story of the elimination of this disease as a public health problem.

This is precisely one of the advantages that we have now to build the story. Those who work in communication know very well that having a good beginning and a good end is equivalent to having 80% of the whole story. And now we are closer to the desired and achievable end.

In spite of this, it is still surprising to see that, in Latin America, where most cases of this disease are concentrated, and whose elimination should be a major political cause in health matters, it is still difficult for it to be recognised as such a cause. And therefore, in society in general, except in areas of high endemicity, it is not a common matter of concern, and knowledge of the disease is far from that of other diseases such as Zika, dengue or Chikungunya. CD is not in the public daily dialogue of the Latin American population, and therefore, it will be difficult to reach that level of knowledge in all the continents where it is widespread.

In my case, the first time I heard about CDs was in the mid-nineties, in Nicaragua, a country of which I am a national. It was at the funeral of a child, in one of the peri-urban neighborhoods of Managua. While accompanying the cortege behind the small coffin, someone commented that the child had suffered a sudden death, and that the hospital could not identify the cause. “It must be Chagas”, someone said spontaneously. It was no more than a sentence without further response and said with the tone of something inevitable, of a cause against which there was nothing to do. No one knew very well what that meant.

Later, I again encountered the worst face of Chagas in the death of another child. He was only nine years old. He was the only son of a friend of mine, a doctor of gynecology. The boy used to spend weekends at a farm owned by his paternal family, and it may have been there that he became infected. There was no diagnosis. One day he began to suffer severe abdominal pains and was rushed to the pediatric hospital. It was appendicitis, they said, and when they went to start the surgery, the little boy could not resist the anesthesia and died in the operating room. The autopsy revealed a swollen heart, huge for the child’s age. They said it was caused by a CD. On the night of the vigil, I still have the image of his mother putting the toys she still kept from when he was younger, inside the small coffin. For me, Chagas had also become inevitable, sinister. That was until I learned about a project of Doctors Without Borders (Médecins Sans Frontières, MSF) in the country, which provided diagnosis and treatment in endemic areas. It was the first time I had known it was treatable.

**Searching for the words and images. The building of a storytelling of Chagas disease**

After that, I dedicated by chance several years of work to MSF, starting with a campaign for access to health in the Americas. In that campaign, one of the main examples was CD. And with several of the organisation’s communicators, we began to think of creative ways to publicise it. One of the activities was the edition of a book of testimonies and quality images that was published in Bolivia and Spain.^1^

For this book, we thought of having the signature of a prestigious writer in the region, such as Eduardo Galeano. He responded to our request, not with an essay or an article, but with a poem. And it was the text that illustrated the flaps and back cover of the book:

*It does not explode like a bomb, nor sound like a shot.*

*Like hunger, it kills silently.*

*Like hunger it kills the silent,*

*those who live condemned to be silent*

*and die condemned to oblivion.*

*Tragedy that rings no bells,*

*patients who do not pay,*

*a disease that does not sell.*

We did not believe that a poem was an innovative format, far from it, but once again we were able to prove the power of words when it comes to symbolise in a story-message the invisible and silent of a health problem. The result was that this poem, even today, continues to be used in thousands of presentations and congresses to illustrate scientific inputs.

When we look closely at the messages associated with CD, we see, as in the previous poem, that a certain grave and tragic tone persists, as in the letters collected by Dias at the beginning of the knowledge of the disease, when there was no treatment. In many of the messages from a pioneering humanitarian organisation such as MSF, there was also a tone of alarm, of urgency, especially in terms of access to diagnosis and treatment. Although efforts in vector control or prevention of blood transfusional transmission have yielded great results, these organisations, together with many other people and experts, considered that the center of all action, the people affected, could not be neglected, as Dr Chagas had already considered.

**Cinema and photography**

From various areas of the world of art and communication, pieces of great merit and value have been generated. In cinema, for example, *Casas de fuego* (Houses of fire), from 1995, which portrays the life and struggle of Salvador Mazza, another of the pioneer physicians and researchers who became involved with CD in Argentina.^2^ And in documentary cinema there have been approaches from many angles. In my case, I had the opportunity to collaborate with a series entitled *Invisibles*, from 2007, which had the support of MSF and the actor Javier Bardem, in which the Spanish director Isabel Coixet approached CD from the perspective of a migrant woman, a necessary angle for a look at a global disease.^3^

Undoubtedly, audiovisual content can contribute greatly to the expansion of knowledge and awareness of CD. The image projected through photography of an invisible disease is one of the most challenging tasks, and those who have tried it know it well. In the 100th anniversary of the discovery of the disease, between 2009 and 2010, many activities were carried out including photographic exhibitions such as those made by Juan Carlos Tomasi and Anna Surinyach for MSF.^4^ Later on, we had the interesting work of the Uruguayan photographer Ana Ferreira, the only photographer, as far as I know, who has specialised in this disease. For her work, she lives with the communities for long periods of time and her camera only shoots when she feels that her gaze has become part of the place and the people, and has matured.^5^

**Information, education and communication (IEC) materials**

It is worth highlighting the enormous efforts made by groups of scientists, communicators and artists in the Oswaldo Cruz Foundation (Fiocruz) or National Scientific and Technical Research Council (CONICET), or the group *De qué hablamos cuando hablamos de Chagas*, in Argentina, which have produced key materials on communication and Chagas, including audiovisuals, and even, recently, a manual of advice and good practices when dealing with the communication of this disease.^6^

There is a wide range of IEC’s materials around the world. However, it is not possible for a single image to portray a multi-causal problem that is not well portrayed. And it is difficult to choose where to focus: rural or urban, poverty or population movements, transmission routes or the complexities of care, endemic or non-endemic areas, and a long etcetera in which each of the components offers interesting angles and stories.

**Communication campaigns**

For that reason, many communication campaigns are limited in scope. But some have managed to have regional or global impacts, such as No Baby with Chagas.^7^ Another communication tool is the contribution of the image of celebrities as it has been explored for Chagas-related campaign spots. The participation of sports or artistic celebrities can be very stimulating. In Chagas, we have even counted on the participation of a star like Messi.^8^ However, the core message, the storytelling, remains the most important thing.

From comics, radio soap operas, songs, playful and informative spots, multilingual posters, pictograms or infographics, many efforts have been carried out, sometimes very fragmentarily.

**And the words again**

The word has also allied with the image to face the challenge of the silence imposed by this disease. In the United States of America, *The kissing bug* was published in 2021, an essay written by a journalist, Daisy Hernández, a pioneering book that provides a view beyond the science, from the Anglo-Saxon world, by someone who had already published in global magazines on issues related to CD.^9^

In the media, pressured by the news rush, CD does not usually represent interesting news, except for the social side and the scientific approach, but this happens very rarely. We have counted on the openness of a global media in Spanish, such as El País, to share the information generated by this disease, but above all to tell “human” stories, as is known in journalism, those kinds of approaches that prioritise the human side of a public health problem.^10^ ^,^ ^11^ It is always said that a single story is worth a thousand stories. The problem is which one to choose.

Although we said before that communication in Chagas has traditionally been tinged with a slightly somber color, or focused on hardship and oblivion, today we have made progress in public health and with tools and knowledge that place us in another historical moment. This must be incorporated into the story-message that is gradually being built.

**The symbol of the World Day and the collective storytelling**

And for this to happen, it is essential that the people affected are the ones who take center stage and have a voice in these messages. The decision to fight for the approval of World Chagas Disease Day undoubtedly brought with it a greater concern for the construction of the narrative-message. I was fortunate to accompany the work of the FINDECHAGAS Assembly, in Mexico, in 2018, when among all of us we bet on finding symbols (images and words) that would identify the global cause of CD. There, thanks to the collaboration of an artist from ConcienciArte in Brazil, the colors green and brown (hope and earth; tree trunk and leaves) were chosen. In addition, a plan for the joint construction of a global logo was proposed.

To do this, we used the path taken especially by the associations of people with HIV, which, as the previous article shows, were pioneers in the progress of the social struggle of people affected by infectious diseases. People with HIV came up with something as simple and powerful as a “red ribbon” to symbolise their struggle. And that was the challenge for Chagas.

The story-message that has been built has one of its milestones in the approval of the World Day that was achieved in 2019, thanks to an unprecedented cross-cutting collaboration that left us lessons such as those set out in the preceding article.

The World Day is one of those symbols needed to break invisibility and silence. But we still need the images and words. The WHO joined this effort, from the Department of Control of Neglected Tropical Diseases, which opened a collaborative platform for communication exchange between affected people, organisations and institutions interested in contributing visions for the construction of these symbols. Each year it is open to proposals for the slogan-message centered on World Day. And it has also served as an exchange of logo proposals.

In this year, 2022, a final logo proposal is presented for the first time and will be disseminated with the support of the Beatchagas platform, organisations of the Chagas Global Coalition, and of course the International Federation of Associations of People Affected by Chagas Disease (FINDECHAGAS). And now, a new campaign is being developed for personalities from the world of sports, art and science to wear the World Chagas Disease Day T-shirt, with the final design of the logo built collectively.^12^

We have yet to find the message-story, the complete storytelling that will have a global impact and lead the narrative to tell the end of this story. But all these advances, fruits of the collective construction of the story (the best stories of humanity were made this way) make us keep the certain hope of being on the verge of achieving it, of being close to those precise images and words that tell the end of Chagas as a public health problem.

Comments on the article: de Oliveira Jr WA, Gómez i Prat J, Albajar-Viñas P, Carrazzone C, Kropf SP, Dehousse A, et al. How people affected by Chagas disease have struggled with their negligence: history, associative movement and World Chagas Disease Day. Mem Inst Oswaldo Cruz. 2022; 117: e220066.
